# ESR Essentials: lung cancer screening with low-dose CT—practice recommendations by the European Society of Thoracic Imaging

**DOI:** 10.1007/s00330-025-11910-9

**Published:** 2025-08-23

**Authors:** Marie-Pierre Revel, Jurgen Biederer, Arjun Nair, Mario Silva, Colin Jacobs, Annemiek Snoeckx, Mathias Prokop, Helmut Prosch, Anagha P. Parkar, Thomas Frauenfelder, Anna Rita Larici

**Affiliations:** 1 Department of Radiology, Cochin Hospital, Université Paris Cité, Paris, France; 2 Department of Diagnostic and Interventional Radiology, University Hospital of Heidelberg, Heidelberg, Germany; 3 Translational Lung Research Center Heidelberg (TLRC), Member of the German Lung Research Center (DZL), Heidelberg, Germany; 4 Faculty of Medicine, University of Latvia, Riga, Latvia; 5 Faculty of Medicine, Christian-Albrechts-Universität zu Kiel, Kiel, Germany; 6 Department of Radiology, University College London Hospital, London, UK; 7 Division of Medicine—Respiratory Medicine, University College London, London, UK; 8 Scienze Radiologiche, Department of Medicine and Surgery (DiMeC), University of Parma, Parma, Italy; 9 Department of Medical Imaging, Radboud University Center, Nijmegen, The Netherlands; 10 Department of Radiology, Antwerp University Hospital, Edegem, Belgium; 11 Department of Radiology, University Medical Center Groningen, Groningen, The Netherlands; 12 Department of Biomedical Imaging and Image-Guided Therapy, Medical University of Vienna, Vienna General Hospital, Vienna, Austria; 13 Department of Radiology, Haraldsplass Deaconess Hospital, Bergen, Norway; 14 Department of Clinical Medicine, Faculty of Medicine and Dentistry, University of Bergen, Bergen, Norway; 15 Institute of Diagnostic and Interventional Radiology, University Hospital Zurich, University of Zurich, Zurich, Switzerland; 16 Department of Radiological and Hematological Sciences, Catholic University of the Sacred Heart, Rome, Italy; 17 Department of Diagnostic Imaging and Oncological Radiotherapy, Advanced Radiology Center, “A. Gemelli” University Polyclinic Foundation IRCCS, Rome, Italy

**Keywords:** Lung cancer, Lung neoplasms, Screening programs (Diagnostic), Artificial intelligence, Multidetector computed tomography

## Abstract

**Abstract:**

Low-dose CT screening for lung cancer reduces the risk of death from lung cancer by at least 21% in high-risk participants and should be offered to people aged between 50 and 75 with at least 20 pack-years of smoking. Iterative reconstruction or deep learning algorithms should be used to keep the effective dose below 1 mSv. Deep learning algorithms are required to facilitate the detection of nodules and the measurement of their volumetric growth. Only large solid nodules larger than 500 mm^3^ or those with spiculations, bubble-like lucencies, or pleural indentation and complex cysts should be investigated further. Short-term follow-up at 3 or 6 months is required for solid nodules of 100 to 500 mm^3^. A watchful waiting approach is recommended for most subsolid nodules, to limit the risk of overtreatment. Finally, the description of additional findings must be limited if LCS is to be cost-effective.

**Key Points:**

- *Low-dose CT screening reduces the risk of death from lung cancer by at least 21% in high-risk individuals, with a greater benefit in women.*
- *Quality assurance of screening is essential to control radiation dose and the number of false positives.*
- *Screening with low-dose CT scans detects incidental findings of variable clinical relevance, only those of importance should be reported.*

## Key recommendations (high level of evidence for all)

- Modern image reconstruction algorithms are needed to keep the effective dose below 1 mSv.
- Nodule management software should be used for improving nodule detection and providing reliable volumetric detection of growth.
- Long-term surveillance of subsolid nodules is safe and limits the risk of overtreatment.

## Introduction

Lung cancer (LC) remains the leading cause of cancer-related mortality worldwide [1].

Immunotherapy and targeted therapy have emerged as an effective but costly way of increasing life expectancy in some patients with advanced tumor stages. Despite these advancements in treatment, the prognosis for LC patients at later stages has only modestly improved over the last decades, highlighting the critical need for effective early detection strategies. Large randomized controlled trials have shown that screening with low-dose computed tomography (LDCT) in at-risk smokers and former smokers can detect LC at a curable stage, thereby reducing lung cancer-related mortality [2, 3]. Improved survival and a stage shift toward stage I LC have been observed following the introduction of lung cancer screening (LCS) in the US [4]. A 2022 Cochrane review of 91,122 participants from eight trials showed that LDCT could reduce LC mortality by at least 21%, with greater benefit in women [5]. Based on this compelling evidence, the European Council revised its 2003 cancer screening recommendations at the end of 2022 to include LC, urging countries to assess the feasibility and effectiveness of LCS [6]. LCS is gradually being introduced in Europe, through projects such as the EU4Health SOLACE (strengthening the screening of lung cancer in Europe) project, under Europe’s Beating Cancer Plan. This manuscript aims to highlight the key elements of LCS for non-thoracic radiologists.

## Eligibility criteria and intervals of LCS

**Fig. 1:**
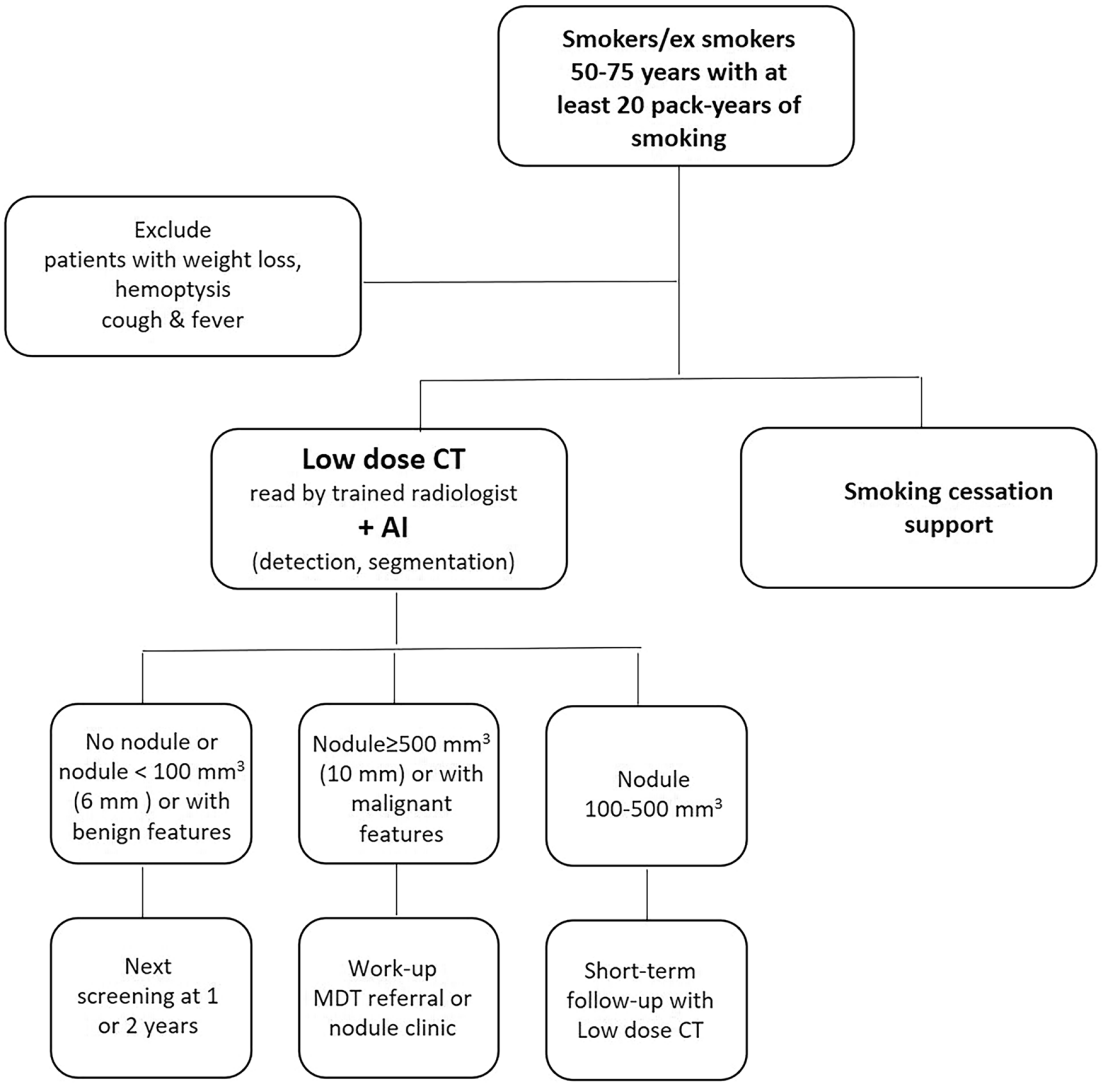
Flowchart summarizing the clinical pathway for LCS

The flowchart summarizing the clinical pathway for LCS is shown in Fig. 1. The benefit of LCS has been demonstrated for individuals at risk according to their age and exposure to smoking. In the absence of validated risk prediction models in Europe, LCS cannot be recommended for never smokers [7]. Table 1 presents the eligibility criteria.

**Table 1:** Radiology essentials for LCS: eligibility criteria

RADIOLOGY ESSENTIALS FOR LCS
Eligibility criteria
• Current and former smokers • Age 50/55 to 74/75 years • 15–20 pack-years • Quit smoking: no more than 10 years or 15 years • Consider use of prediction models
Intervals of LCS
• Annual screening • Consider biennial screening after a negative scan
Technical requirements—acquisition modalities
• Unenhanced thoracic CT in full inspiration • Multidetector CT with 32 or more rows • Gantry rotation time ⩽ 0.5 s • Effective radiation dose < 1 mSv • CTDIvol 0.4 mGy, 0.8 mGy, and 1.6 mGy (weight < 50, 50–80 kg, and > 80 kg) • Iterative or deep learning reconstruction • Use of ultra-low-dose CT protocols is being investigated
Reading modalities and the role of AI software
• Reading by radiologists trained in LCS • Use of 10 mm to 15 mm MIP images • Volumetry software • Consider AI as a second or concurrent reader
Criteria for positive, negative, or indeterminate screen results
• ESTI nodule management guidelines for solid nodules: < 100 mm^3^ negative, > 500 mm^3^ diagnostic work-up, between these values re-evaluation and volumetric doubling time measurement • Long-term surveillance of subsolid nodules is safe • Recognition of typically benign lesions (e.g., intrapulmonary lymph nodes and hamartomas) to reduce unnecessary follow-up
Incidental findings relevant to the report
• Most incidental findings are benign and should not be systematically reported • Consider reporting very suspicious findings and severe coronary artery calcification
Reporting
• Structured reporting with ESTI template

The lower and upper age limits at which screening is usually recommended is between 50–55 years and 74–75 years, respectively, as these were the age ranges in most LCS studies [8]. The United States Preventive Services Task Force recommendations have recently extended the upper limit to 80 years [9]. The exclusion of older (over 65) people who have quit smoking for more than 10 years or 15 years is now increasingly questioned, since ageing increases the risk of LC [10]. The cumulative cigarette consumption varied in the published studies from 15 pack-years (PY) in the Depiscan study [11] to 30 PY in the National Lung Screening Trial (NLST) study [2], and has been set at 20 PY in the USA [9]. Comprehensive risk models (including family history of LC, asbestos exposure, etc.) were proposed to optimize the inclusion of milder smokers with further risk factors. The prostate, lung, colorectal, and ovarian cancer risk-prediction model 2012 is currently the most widely used [12]. Machine learning models that take into account clinical and/or biological parameters have recently been proposed, and await validation [13, 14].

A number of potential blood, exhaled air, or sputum biomarkers are being studied, but none have yet been validated to help detect LC at an early stage [15]. Rather than being used as a first-line selection method, they could be combined with LDCT to increase specificity, if their cost is acceptable [16].

It was conventionally assumed that LCS with LDCT had to be undertaken annually, but a biennial screening interval is increasingly proposed as more sustainable, since it is safe after a negative scan [17]. A longer interval is associated with a higher risk of interval cancers. Further refinement of selection and screening strategy is continuously being investigated; the European 4-IN THE LUNG RUN trial aims to investigate personalized invitation and frequency of LCS [18].

Participation is key and is mainly driven by the personal perceived risk of lung cancer [19].

## Technical requirements

The prerequisite for efficient and reliable LCS is thin-slice volumetric CT scans. A multidetector CT with 32 or more detector rows with a gantry rotation time of ⩽ 0.5 s is needed for a complete chest coverage in < 10 s breath-hold at full inspiration in the supine position. Images should be reconstructed at a slice thickness of 1.0 mm or less (preferred ⩽ 0.75 mm). Overlapping reconstructions are not mandatory. The field of view should be adapted to full lung parenchyma coverage, without including the whole chest wall. Tube voltage and current are adjusted to patient morphology with the aim of keeping the average effective radiation dose below 1 mSv (milliSievert). CT dose index volumes of 0.4 mGy, 0.8 mGy, and 1.6 mGy (milligray) are recommended for participants < 50, 50–80, and > 80 kg, respectively. Available means to reduce radiation exposure include automatic exposure control, organ dose modulation, and prefiltration. Iterative or deep learning reconstruction should be used for noise reduction instead of filtered back projection. Ultra-low-dose protocols resulting in an effective dose close to that of a frontal and lateral chest-x ray (0.13 mSv) have not been validated prospectively in the context of LCS. Notably, acquisition and reconstruction parameters should be kept constant for the volumetric follow-up of indeterminate lung nodules.

A detailed description of the LCS technical requirements can be downloaded from the European Society of Thoracic Imaging (ESTI) website [20].

## Reading modalities and the role of AI software

The quality of the reading of LDCT images is a crucial component in an LCS program and should involve radiologists with an appropriate level of training and expertise. Qualification of readers can be achieved with appropriate training programs, such as the ESTI LCS Certification Project [20]. Visual reading is complemented by coupled reading of thin slab axial images and 10–15 mm maximum intensity projections (MIP), to maximize detection of solid nodules. Volumetry software has been used in the NELSON and other European trials and is the recommended method for nodule measurement in the European Position Statement (EUPS) guidelines on LCS [21]. Volumetry is more accurate and reproducible than diameter measurement for solid pulmonary nodules. On the other hand, the reliability of volumetry tools has not been well studied in subsolid nodules (SSN) and thus requires further research.

Artificial intelligence (AI) has the potential to increase the efficiency of LCS. AI software has much improved in the past decade as a result of the revolution in AI technology, and is therefore expected to play an increasingly important role for detection, volumetry, characterization, and malignancy risk estimation of pulmonary nodules. At present, there are more than 10 CE (conformité Européenne) marked commercially available products in Europe, which have been cleared for use as an aid to the radiologist. The application of AI products for nodule detection has the potential to increase the sensitivity for pulmonary nodule detection and reduce reading time. New algorithms are now also available for the estimation of malignancy for lung nodules. Studies have shown that these algorithms achieve comparable or slightly inferior performance compared to specialized radiologists, notably with the risk of false negative for pulmonary mass and mediastinal lesions [22–24]. However, the scientific evidence on the independent performance of most available products is still limited [25]. Future validation studies are needed to investigate how AI products can be best implemented and whether their performance as a second or concurrent reader is comparable to double reading [26].

## Criteria for positive, negative, or indeterminate screen results

To maximize cancer detection while minimizing harms from over-investigation, nodules are referred for further investigation only when there is a high probability of malignancy, based on size and non-size characteristics, such as spiculations, pleural indentation, or cavitation with thick walls.

For baseline, the size threshold for a solid non-calcified nodule according to the EUPS guidelines is a volume of 300 mm^3^ [21]. The British Thoracic Society has further modified this, with referral only when the malignancy risk using the Brock model is at least 10%, thus incorporating other important nodule features (spiculation, upper lobe location, and nodule count) [27].

**Fig. 2:**
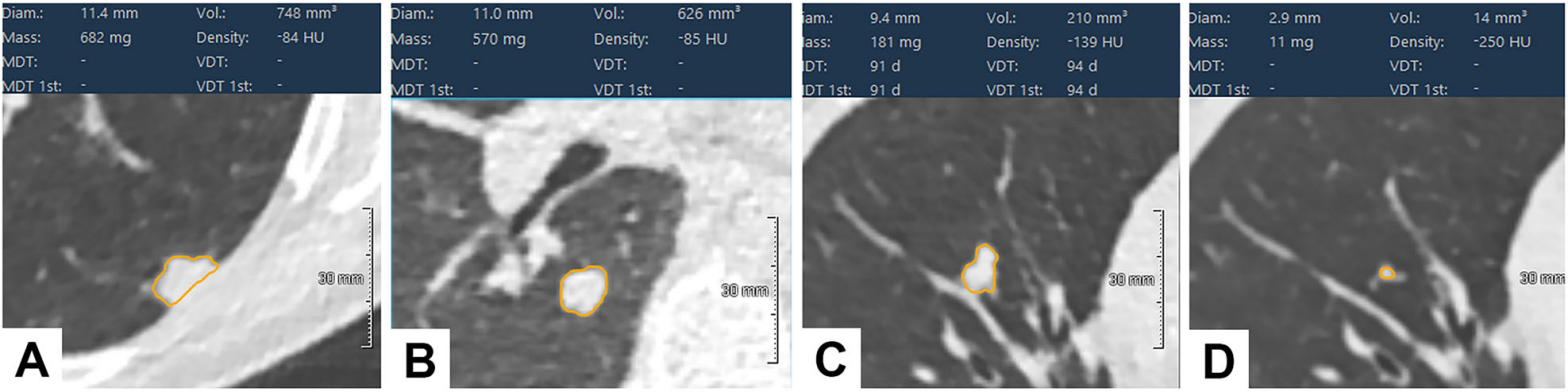
High-risk solid nodules. Axial LDCT images from different patients. **A**, **B** correspond to two different patients, and **C**, **D** correspond to a third patient with two studies. **A** A pleural-based solid nodule in the left lower lobe, with a volume of 748 mm^3^ at baseline. Positive FDG-PET led to a percutaneous biopsy confirming malignancy. A pT2 lung adenocarcinoma invading the visceral pleura was resected. **B** A 626 mm^3^ solid nodule in the right Nelson segment was found at baseline. The nodule was negative on PET and remained stable on follow-up CT scan. **C** A solid nodule of 210 mm^3^ in the middle lobe was discovered at one year follow-up, which already existed on the baseline scan (**D**). VDT was only 94 days. An 11 mm squamous cell carcinoma was found at the time of surgery

The ESTI guidelines have been inspired by these existing guidelines, but their primary objectives are to reduce the number of follow-up examinations while avoiding the risk of a major change stage shift, and the overdiagnosis risk (defined below) [28]. High-risk solid nodules are those with a large volume and/or a fast volume doubling time (VDT) (Fig. 2).

At baseline, the high-risk size threshold is ≥ 500 mm^3^, in line with NELSON’s original criteria (or a baseline maximum diameter of ≥ 10 mm if volumetry is not available or fails), requiring referral to a multidisciplinary team (MDT) for further diagnostic work-up. Solid nodules with a baseline volume of less than 100 mm^3^ represent a negative screen result. Solid nodules between 100 mm^3^ and 250 mm^3^ require a 6-month follow-up and referral to MDT if their VDT is < 400 days, whereas those between 250 mm^3^ and 500 mm^3^ are reevaluated at 3 months and referred to MDT if their VDT is < 250 days. For those resolving or with slow growth, the following LDCT may be scheduled 12 months after the last CT scan, in order to minimize radiation and optimize the number of patient visits.

At annual follow-up, a VDT < 500 days is considered suspicious. If volumetry fails, an increase in diameter > 1.5 mm is considered to indicate significant growth.

New nodules are usually inflammatory, and most (66% in the NELSON trial) will resolve in the next follow-up CT.

**Fig. 3:**
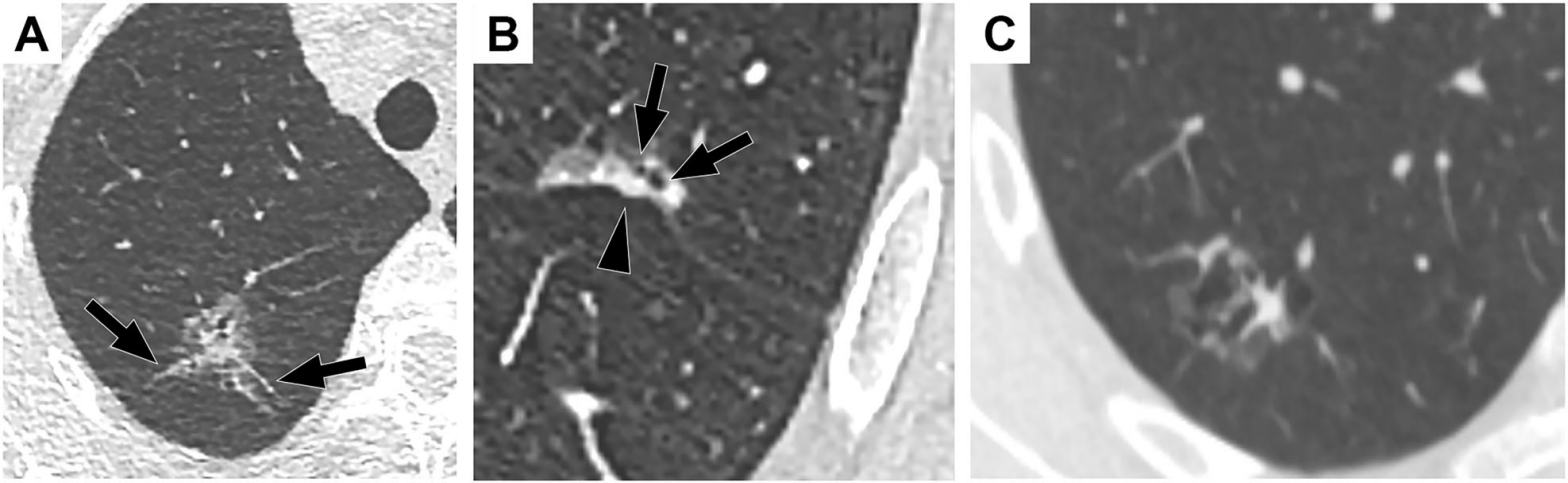
Suspicious morphological signs. **A** Part-solid nodule with spiculations (arrows). **B** Part-solid nodule with pleural indentation (arrowhead) and bubble-like lucencies (arrows). **C** Multilocular cyst

Those ≥ 30 mm^3^ require short-term (3-months) re-evaluation and are referred to MDT if they grow. SSN include pure ground glass and part-solid nodules, considered a negative result if they measure < 3 cm and have a solid component < 6 mm, unless they present with suspicious morphology signs (Fig. 3). Those with a large solid component (≥ 1 cm) at baseline require 1-month re-evaluation and indicate a positive screen result unless they spontaneously resolve.

**Fig. 4:**
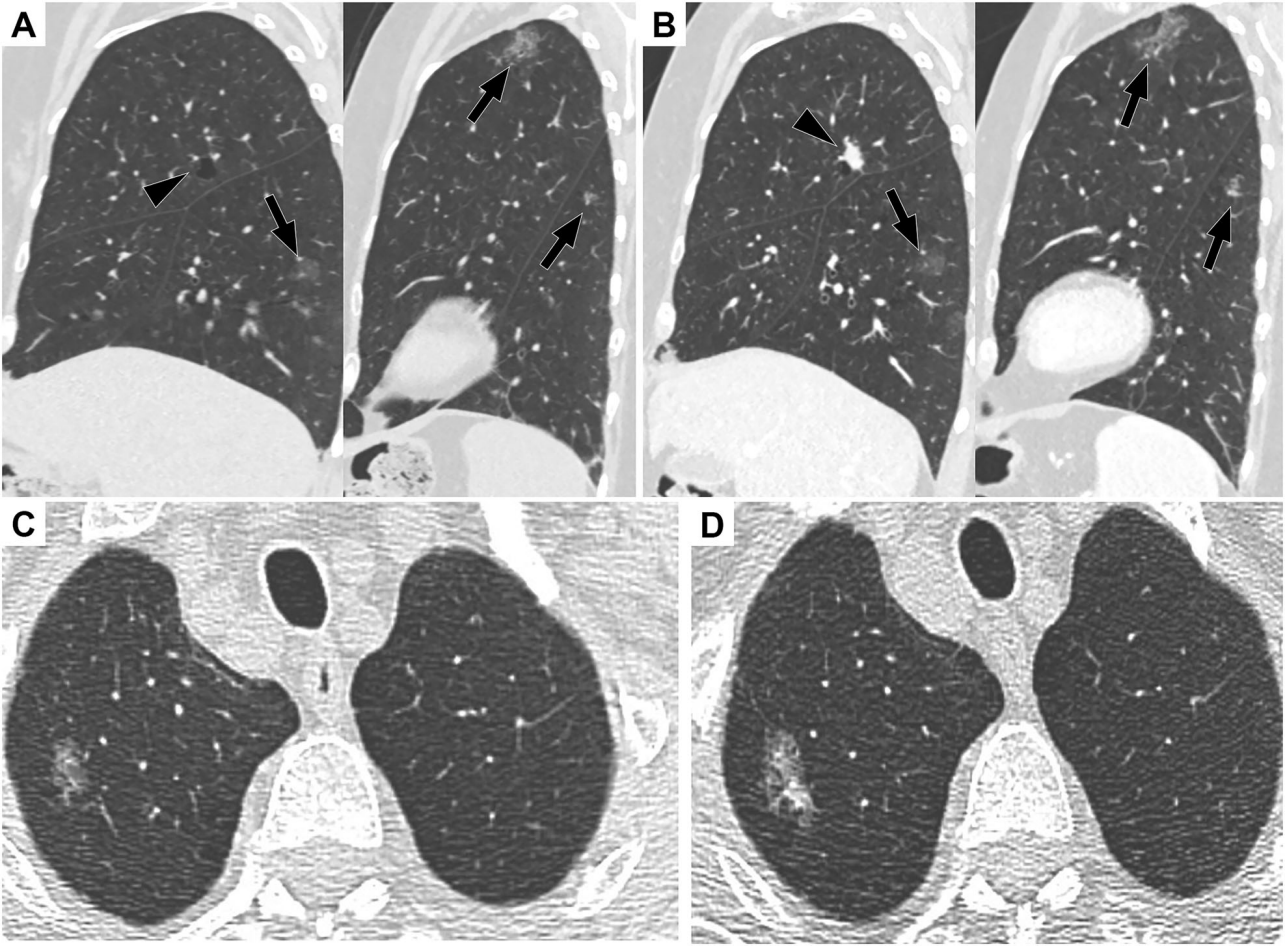
CT follow-up in a patient with subsolid nodules. **A** Pure ground glass nodules of the right lower lobe and the left upper and lower lobes (arrows). **B** Four years later (standard dose contrast-enhanced CT), the pure ground glass nodules were unchanged, whereas a spiculated solid nodule corresponding to an invasive adenocarcinoma had developed in the lung cyst wall (arrowhead). **C** A pure ground glass nodule was found in the apical segment of the right upper lobe. **D** Three years later, the nodule had grown and developed a solid portion posteriorly

Persisting subsolid nodules need to demonstrate growth in their solid component (Fig. 4) or altered morphology (e.g., the development of bubble-like lucencies) as criteria for positivity. Complex cysts such as multilocular cysts and LC associated with cystic airspaces represent a specific category to identify, and are usually related to invasive forms of LC [29] (Fig. 3).

## Subsolid nodules and the risk of overdiagnosis

In the vast majority of cases, persistent SSN represents indolent forms of lung adenocarcinoma, which progressively evolve from atypical adenomatous hyperplasia to in situ proliferation, then to minimally invasive forms and finally to invasive forms of adenocarcinoma. They represent the main source of overdiagnosis during LCS.

Overdiagnosis and overtreatment refer to the screen detection and treatment of cancer that would not have affected the subject’s life expectancy because of low tumor aggressiveness (which is the case of SSN) or concurrent comorbidities, which are most frequent in advanced age. Long-term surveillance of SSN to detect growth of solid component (either new or increasing) has proven to be a safe strategy [30]. Indeed, individuals with SSN are more likely than others to develop LC, but clinically relevant LC mainly develop at another location [31] (Fig. 4). Those arising from the SSN were never the cause of death over a median follow-up of 9 years in a series evaluating 400 SSN from the Multicentric Italian lung detection (MILD) trial [30]. Such results were subsequently confirmed in a clinical setting and the bioMILD LCS trial [31, 32].

## Intrapulmonary lymph nodes (IPLNs) and other benign findings

**Fig. 5:**
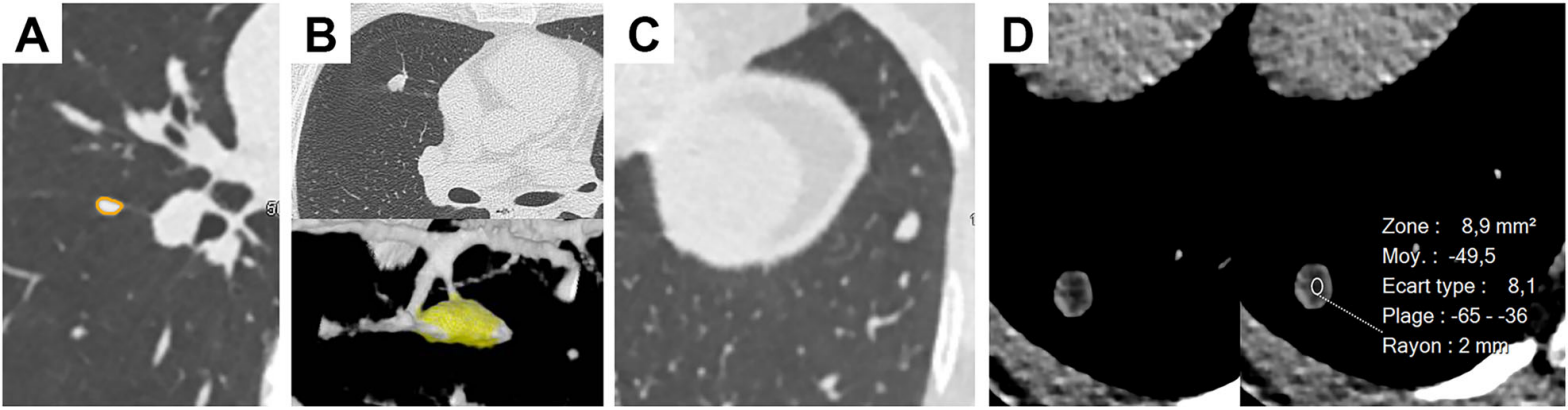
Solid nodules with benign features. IPLNs, perifissural opacities (PFOs), and fat-containing nodules. **A** PFO within the right major fissure. **B** PFO with a beret shape in contact with the minor fissure. **C** IPLN in the subpleural location of the left lower lobe. **D** Fat-containing nodule. The measured attenuation was −49 HU, with a standard deviation of only 8 HU, which allowed us to be certain about the fat content

Pulmonary nodules are extremely common, and those with benign features should not be investigated further. They are illustrated in Fig. 5.

IPLNs are a very frequent cause of such nodules. They meet the following criteria: a size of less than 12 mm, a distance of less than 15 mm from the pleura, and a location below the level of the carina [33]. They have sharp margins and usually a round, oval, or polygonal shape. On 3D reconstructions, they appear to have a flat side, which represents the hilum of the node. Perifissural opacities (PFOs) represent a variant where IPLNs are in close contact with a fissure. Most IPLNs and PFOs have a volume of less than 100 mm^3^, which corresponds de facto to a negative screening result, but they are likely to show fluctuations in volume over time.

Fat-containing nodules correspond to hamartomas, a benign cause of solid nodules, whose diagnosis is definitive, provided that the attenuation measurement is rigorously performed on the mediastinum filter without including lung parenchyma and with a narrow standard deviation (Fig. 5). Calcifications can also be a sign of benignity, although some malignant nodules may contain calcifications. Only central calcifications, according to multiplanar evaluation, should be considered a reliable sign of benignity [34].

## Incidental/additional findings relevant to the report

Incidental findings (IFs) are unexpected results from LDCT scans that could impact a patient’s health but are not related to LC detection. Commonly encountered IFs include pulmonary, cardiovascular, and gastrointestinal findings [35]. Most are benign and have little clinical significance [36]. The variability in reporting prevalence, ranging from 1% to 94%, underscores the absence of standardized procedures for reporting and managing IFs [35].

LDCT is not a suitable technique for the evaluation of solid organs such as the liver or kidney, which are only partially evaluated. This also applies to the breast, which is not entirely included in the field of view. Furthermore, the wide standard deviation of the attenuation measurement makes the assessment of the cystic or solid nature of the anomalies unreliable. Thus, it cannot be recommended to systematically assess these organs, which have important legal implications. Of course, very suspicious findings need to be reported, which is left to the radiologists’ personal judgment.

Regarding additional findings to report, evaluating coronary artery calcifications might be relevant, as smoking is a common risk factor for LC and coronary artery disease. I-ELCAP recommends using the Shemesh score, which is predictive of death from cardiovascular disease [37]. Similarly, reporting severe aortic valve calcification or aortic aneurysm might be relevant, even though precise guidelines are still lacking. By incorporating evidence-based strategies, including automatic detection and data-mining tools powered by AI, LCS programs will improve in accuracy and cost-effectiveness.

## Reporting: structured report template proposal

The implementation of a structured reporting (SR) has been highly recommended in the European Society of Radiology (ESR) and the European Respiratory Society (ERS) ESR/ERS statement paper on LCS, recognizing its importance for improving the quality, patient outcome, and cost-effectiveness of LCS [38]. The use of an SR format promotes process consistency and facilitates periodic monitoring and auditing, furtherly optimizing the screening program [39]. ESTI proposed a comprehensive but simplified SR of LDCT, which includes demographics, technical details of LDCT acquisition, and nodule characteristics (type, morphology) [20]. Links are provided for computation of the risk of nodule malignancy at baseline (Brock model), evaluation of growth at subsequent LDCT examinations, and management of screen-detected nodules according to international guidelines. In addition, extranodular CT findings are included in the format, such as coronary artery calcification for which workup is suggested, in agreement with the recommendations mentioned above.

**Fig. 6:**
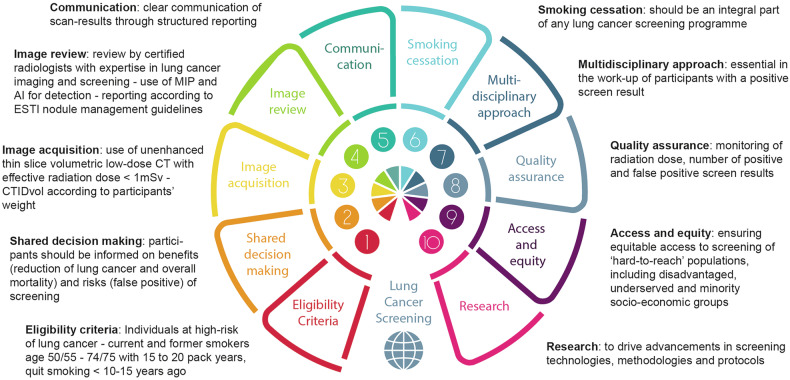
Requirements for a successful LCS program

Figure 6 summarizes the requirements for a successful LCS program.

## Summary statement

LCS applies to smokers and ex-smokers with at least a 20 PY smoking history and is based on LDCT screening, which may be biennial after a negative scan. Recommendations for CT dose index volume are 0.4 mGy, 0.8 mGy, or 1.6 mGy for participants weighing < 50 kg, 50–80 kg, or > 80 kg, respectively. Iterative or deep learning reconstruction is required to reduce noise, as well as the use of CE-marked software for nodule detection and volumetry. Solid nodules ≥ 500 mm^3^ represent a positive screening result, as do thick-walled cavitary nodules, complex cysts, spiculated nodules, or nodules with bubble-like lucencies or pleural retraction. Nodules with benign features or a volume < 100 mm^3^ represent a negative result, and those with a volume between 100 mm^3^ and 500 mm^3^ require reassessment at 3 or 6 months and measurement of their VDT. Long-term surveillance of SSN to detect new or growing solid portions is safe and limits the risk of overtreatment. Severe coronary artery or aortic valve calcifications should be reported, as well as highly suspicious findings other than pulmonary nodules. The description of additional findings should be limited, and evidence-based strategies remain to be defined. AI is set to play an increasingly important role in LCS. Radiologists should take an active role in guiding and optimizing LCS practices.

## Patient summary

LCS is aimed at smokers and former smokers aged 50 and over who have smoked the equivalent of at least one pack of cigarettes a day for 20 years. Screening is based on a low radiation dose CT scan repeated annually or every 2 years if the first scan is negative. Screening is considered positive for nodules that are either initially large, or show rapid growth over time. Scans must be read by trained and experienced radiologists, using modern AI solutions. Today’s knowledge enables optimization of screening by referring only a minority of suspicious nodules for further investigation.

## Abbreviations

AI: Artificial intelligence
CE: Conformité Européenne
ERS: European Respiratory Society
ESR: European Society of Radiology
ESTI: European Society of Thoracic Imaging
EUPS: European position statement
IFs: Incidental findings
IPLNs: Intrapulmonary lymph nodes
LCS: Lung cancer screening
LDCT: Low-dose computed tomography
MDT: Multidisciplinary team
mGy: Milligray
MIP: Maximum intensity projection
mSv: MilliSievert
PFOs: Perifissural opacities
PY: Pack-year
SOLACE: Strengthening the screening of Lung Cancer in Europe
SR: Structured reporting
VDT: Volume doubling time
